# Biochemotherapy for the treatment of metastatic malignant melanoma: a clinical practice guideline

**Published:** 2008-04

**Authors:** S. Verma, T. Petrella, C. Hamm, K. Bak, M. Charette, the members of the Melanoma Disease Site Group of Cancer Care Ontario’s Program in Evidence-based Care

**Affiliations:** * The Ottawa Hospital, Ottawa, ON; † Cancer Care Ontario Program in Evidence-based Care, McMaster University, Hamilton, ON; ‡ Sunnybrook Regional Cancer Center, Toronto, ON; § Windsor Regional Cancer Centre, Windsor, ON

**Keywords:** Melanoma, biochemotherapy, practice guideline

## Abstract

**Questions:**

- What is the role of biochemotherapy in the treatment of metastatic malignant melanoma?
- What are the adverse effects and effects on quality of life of biochemotherapy as a treatment option?

For the purposes of this report, “biochemotherapy” is defined as a therapeutic regimen that includes, at a minimum, chemotherapy (either single-agent or combination) and interleukin-2.

**Perspectives:**

Although early detection, appropriate surgery, and in some cases adjuvant therapy have improved outcomes, at least one third of patients with early-stage melanoma will develop metastases. Recently, in an effort to potentially maximize outcomes, the combination of chemotherapy and immunotherapy (biochemotherapy) was evaluated. The level of interest that this approach has generated, particularly with regard to the apparently high response rates seen in this otherwise devastating illness, was sufficient to merit closer examination by the Melanoma Disease Site Group (dsg) of Cancer Care Ontario’s Program in Evidence-based Care (pebc).

**Outcomes:**

Outcomes of interest include response rate, diseasefree survival, overall survival, quality of life, and incidence of grades 3 and 4 toxicities.

**Methodology:**

Evidence was selected and reviewed by three members of the pebc’s Melanoma dsg and by two methodologists. The present practice guideline report was reviewed and approved by the Melanoma dsg, which comprises medical and radiation oncologists, surgeons, and dermatologists. External review by Ontario practitioners was obtained through a mailed survey, the results of which were incorporated into the practice guideline. Final approval of the original guideline report was obtained from the pebc’s Report Approval Panel.

**Results:**

Clinical recommendations were drafted based on the evidence identified through a systematic review. The practice guideline report with draft recommendations was mailed to Ontario practitioners for external review and to the Report Approval Panel. Feedback from both groups was incorporated into this report to create the final practice guideline.

**Practice Guideline:**

The recommendations that follow apply to adult patients with metastatic malignant melanoma.

Because of the inconsistent results of the available studies with regard to benefit (response, time to progression, and survival) and consistently high toxicity rates, biochemotherapy is not recommended for the treatment of metastatic melanoma.

## 1. QUESTIONS

- What is the role of biochemotherapy in the treatment of metastatic malignant melanoma?
- What are the adverse effects and effects on quality of life of biochemotherapy as a treatment option?

For the purposes of this report, “biochemotherapy” is defined as a therapeutic regimen that includes, at a minimum, chemotherapy (either single-agent or combination) and interleukin-2. Outcomes of interest included response rate, disease-free survival, overall survival, quality of life, and adverse events.

## 2. CHOICE OF TOPIC AND RATIONALE

The incidence of malignant melanoma has been increasing steadily since the end of the 1950s. In 2006, the number of new cases diagnosed in Canada was estimated at 4500, with 880 deaths 1. Of those new cases, 1910 were estimated to have been diagnosed in Ontario, with 430 of the 880 estimated deaths occurring in that province 1.

Although early detection, appropriate surgery, and in some cases adjuvant therapy have improved outcomes, at least one third of patients with early-stage melanoma will develop metastases. The prognosis for patients with metastatic melanoma remains dismal. Numerous clinical trials have attempted to identify potential treatments for those patients, but most have failed to establish any single “best” modality. Systemic approaches that have been evaluated sequentially to date include cytotoxic chemotherapy (single agents and multi-drug combinations) and immunotherapies, including interferon- (ifn) and interleukin-2 (il-2).

Recently, in an effort to potentially maximize outcomes, the combination of chemotherapy and immunotherapy (called “biochemotherapy”) has been evaluated. A number of randomized clinical trials have been completed, and their results have now been reported. The level of interest generated by this approach, particularly with respect to the apparently high response rates seen in an otherwise devastating illness, was sufficient to merit closer examination by the Melanoma Disease Site Group (dsg) of Cancer Care Ontario’s Program in Evidence-Based Care (pebc).

## 3. METHODS

### 3.1 Guideline Development

The present practice guideline report was developed by the pebc, using the methods of the practice guidelines development cycle 2. Evidence was selected and reviewed by three members of the Melanoma dsg and by two methodologists. Members of the Melanoma dsg disclosed potential conflict of interest information.

This practice guideline is a convenient and up-to-date source of the best available evidence on chemotherapy (either single-agent or combination) and il-2 in the treatment of metastatic melanoma. The present report was developed through systematic review, evidence synthesis, and input from practitioners in Ontario. The practice guideline is a companion to a systematic review published elsewhere 3. Both documents are intended to promote evidence-based practice in Ontario, Canada. The pebc is editorially independent of Cancer Care Ontario and the Ontario Ministry of Health and Long-Term Care.

External review is obtained for all practice guideline reports through a mailed survey of Ontario practitioners. The survey consists of items that address the quality of the draft practice guideline report and recommendations, and that ask whether the recommendations should serve as a practice guideline. Final approval of the practice guideline report is obtained from the pebc’s Report Approval Panel.

### 3.2 Literature Search Strategy

A systematic search of the medline, embase, cancerlit, and Cochrane Library databases was conducted. In addition, the proceedings of the annual meetings of the American Society of Clinical Oncology were searched for reports of newly completed trials. Randomized controlled trials (rcts), meta-analyses of rcts, evidence-based clinical practice guidelines, and systematic reviews were eligible for inclusion if they reported on at least one of the outcomes of interest.

## 4. RESULTS

Nine randomized controlled trials 4–13 of biochemotherapy plus one systematic review with meta-analysis 14 were located and included in the systematic review.

Of the nine eligible trials, six compared various regimens of chemotherapy with biochemotherapy 4–9, two compared chemotherapy plus ifn with biochemotherapy 10,11, and one trial compared biochemotherapy with a combination of ifn and il-2 12. Dose and method of administration varied from trial to trial.

Seven of the nine trials reporting on response rate provided statistical comparisons 4,7–13. Only two trials reported statistically significant response rates favouring treatment with biochemotherapy 8,12; five trials failed to detect any significant differences. None of the nine trials detected a statistically significant survival improvement with biochemotherapy.

When data were pooled, biochemotherapy was superior to chemotherapy in response [relative risk (rr): 1.52; 95% confidence interval (ci): 1.24 to 1.87; *p* < 0.0001] and delayed progression at 6 months (rr: 0.85; 95% ci: 0.75 to 0.96; *p* = 0.008), but not in decreased mortality at 12 months (rr: 0.98; 95% ci: 0.84 to 1.16; *p* = 0.85).

One study 15 evaluated quality of life in the patients included in a rct of biochemotherapy versus chemotherapy 7. Overall quality of life significantly declined through the 5th treatment cycle with biochemotherapy (*p* = 0.03).

Biochemotherapy is a toxic therapy, and patients are likely to experience serious hematologic, gastrointestinal, cutaneous, and constitutional toxicities. In addition, there are risks of cardiovascular toxicities such as myocardial events and arrhythmias, hypotension, capillary leak syndrome, hepatotoxicity, and renal toxicity. When treatment is conducted in the correct setting, grades 3 and 4 toxicities appear to be manageable, and treatment-related death can be minimized.

## 5. DSG CONSENSUS PROCESS

The draft guideline and systematic review were approved by the Melanoma dsg in December 2006.

## 6. REPORT APPROVAL PANEL

### 6.1 Results

In January 2007, before the evidence-based report was submitted for external review, the report was reviewed and approved by the pebc’s Report Approval Panel. The Panel consists of two members, including an oncologist with expertise in clinical and methodology issues. These key concerns were raised by the Panel:

- Was there consistency in the biochemotherapy regimens tested in the phase ii trials that led to the reported phase iii trials?
- Might variation in the response rates across the reported trials be related to different tumour response evaluation criteria?
- Was it appropriate to use 12-month mortality data for the meta-analysis, when the report Introduction indicates that median survival for this patient group is 6–8 months?
- Does the Results section show inconsistency in commenting on a survival trend in the Rosenberg trial 3, which suggests some benefit with chemotherapy, but not in the Eton trial 4, which suggests some benefit with biochemotherapy.

In addition, one member of the Panel suggested that explicit statements about “policy-determining” outcomes would be helpful. Because in the present case, the relevant outcomes appear to be overall survival coupled with treatment toxicity, the Panel also suggested a defining statement to indicate that focus.

### 6.2 Modifications/Actions

In response to the Panel feedback, the Melanoma dsg

- acknowledged that an optimum regimen had not been identified for biochemotherapy, and that the regimens used in the phase ii and phase iii trials had varied, generally corresponding with institutional or organizational preferences.
- indicated that the criteria used to define tumour response in most trials were those of the World Health Organization (or a similar definition) and added a brief statement to the Trial Descriptions section of the systematic review to summarize those data.
- agreed that 11–12 months was a reasonable time point for data pooling in meta-analysis, because the median survival for most of the reported trials fell close to that range. In addition, the pooled 6-month survival data showed a result similar to that obtained at 12 months, and that finding was indicated in the Results section of the report.
- acknowledged the need for consistency in data presentation and added a comment on the contrasting results of the Eton trial 4 following the discussion of the Rosenberg trial 3 results in the Results section of the report.
- Finally, although the pebc guidelines are considered in policy determination, the authors considered that their main purpose was to provide guidance for clinicians, and they therefore did not wish to comment on policy-determining outcomes. In developing the recommendations, all relevant outcomes were considered, and the dsg felt that the current wording of the recommendation accurately reflected that fact. The recommendation was therefore not revised.

## 7. PRACTITIONER FEEDBACK

### 7.1 Methods

Following discussion and consensus, the Melanoma dsg circulated the clinical practice guideline and systematic review to clinicians in Ontario for review and feedback.

Feedback was obtained through a mailed survey of 12 medical oncologists in Ontario. The survey consisted of items evaluating the methods, results, and interpretive summary used to inform the draft recommendations and asking whether the draft recommendations should be approved as a practice guideline. Written comments were invited. The survey was mailed on March 5, 2007. Follow-up reminders were sent at 2 weeks (post card) and 4 weeks (complete package mailed again). The Melanoma dsg reviewed the results of the survey.

### 7.2 Results

From among the 12 surveys mailed, 5 responses were received (42% response rate). Responses include returned completed surveys, plus telephone, fax, and e-mail responses. Of the practitioners who responded, 4 indicated that the report was relevant to their clinical practice, and they completed the survey. Table I summarizes key results of the practitioner feedback survey.

### 7.3 Summary of Written Comments

Only 1 respondent provided a written comment, which confirmed agreement with the report.

## 8. PRACTICE GUIDELINE

The present report reflects the integration of feedback obtained through the external review process with final approval given by the Melanoma dsg and the pebc’s Report Approval Panel.

### 8.1 Target Population

The recommendations set out in the practice guideline apply to adult patients with metastatic malignant melanoma.

### 8.2 Recommendations

Because of the inconsistent results of the available studies with regard to benefit (response, time to progression, and survival) and consistently high toxicity rates, biochemotherapy is not recommended for the treatment of metastatic melanoma.

## 9. PRACTICE GUIDELINE DATE

Completed April 2007. Practice guidelines developed by the pebc of Cancer Care Ontario are reviewed and updated regularly. Please visit the pebc section of the Cancer Care Ontario Web site (www.cancercare.on.ca/index_practiceGuidelines.htm) for the full guideline report and subsequent updates.

## Figures and Tables

**TABLE I:** Practitioner responses to eight items on the practitioner feedback survey

Item	Strongly agree or agree	[n (%)] Neither agree nor disagree	Strongly disagree or disagree
The rationale for developing a guideline, as stated in the “Introduction” section of the report, is clear.	4 (100)	0	0
There is a need for a guideline on this topic.	3 (75)	1 (25)	0
The literature search is relevant and complete.	4 (100)	0	0
The results of the trials described in the report are interpreted according to my understanding of the data.	4 (100)	0	0
The draft recommendations in the report are clear.	4 (100)	0	0
I agree with the draft recommendations as stated.	4 (100)	0	0
This report should be approved as a practice guideline.	4 (100)	0	0
	*Very likely or likely*	*Unsure*	*Not at all likely or unlikely*
	
If this report were to become a practice guideline, how likely would you be to make use of it in your own practice?	4 (100)	0	0
